# PDGF, NGF, and EGF as main contributors to tumorigenesis in high-risk retinoblastoma

**DOI:** 10.3389/fonc.2023.1144951

**Published:** 2023-10-30

**Authors:** Karim Al-Ghazzawi, Michael Wessolly, Sami Dalbah, Petra Ketteler, Tobias Kiefer, Nikolaos Bechrakis, Jabbarli Leyla, Saskia Ting, Eva Biewald, Fabian D. Mairinger

**Affiliations:** ^1^ Department of Ophthalmology, University Hospital Essen, Essen, Germany; ^2^ Department of Pathology, University Hospital Essen, University of Duisburg-Essen, Essen, Germany; ^3^ Department of Pediatric Hematology and Oncology, University Hospital Essen, University of Duisburg-Essen, Essen, Germany; ^4^ Institute of Pathology Nordhessen, Kassel, Germany

**Keywords:** retinoblastoma - epigenetics, retinoblastoma, PDGF = platelet-derived growth factor, MAPK/ERK2 pathway 1, pediatric cancer and oncology, biomarkers, targeted therapy, growth factors (angiogenesis factors)

## Abstract

Retinoblastoma (RB) is the most common form of eye cancer experienced in childhood. Its aggressive malignancy is associated with excellent survival rates in high-income countries; however, the prognosis in third-world countries is less favorable. Early diagnosis can maximize the patient’s visual outcomes and their survival rate. Therapy should be conducted in highly specialized treatment centers. Intravenous chemotherapy (IVC) in bilaterally affected children currently forms the majority of therapy. Local destructive procedures and local chemotherapies such as intra-arterial chemotherapy (IAC) or intravitreal chemotherapy can be taken into consideration depending on the extent and size of the tumor. Nonetheless, children and parents remain under constant stress, revisiting doctors for medical treatment and fearing vision loss and even enucleation of the eye. Adequate molecular patient stratification to improve targeted therapy is still lacking. This retrospective study analyzed formalin-fixed paraffin-embedded specimens from a cohort of 21 RB samples. A total of 11 of those samples showed undifferentiated retinoblastoma (URB) histopathological risk features, and the other 10 showed differentiated retinoblastoma (DRB) histopathological grading. RNA from all samples was isolated and analyzed via digital gene expression patterns. Conductors of cell survival and DNA repair were dominant in the DRB samples. In contrast, the agents responsible for cell–cycle progression and apoptosis were overexpressed in URB samples. Our work reveals the importance of molecular mechanisms within the immune system subjected to histologic subtypes of RB, providing more detailed background on their genetic behavior. This is of great interest for therapeutic strategies, such as targeted immune- and gene-based therapies, for retinoblastoma.

## 1 Introduction

Retinoblastoma (RB) is the most common primary intraocular malignancy in children. It has an incidence rate of 1 in 14,000–20,000 live births and has no gender predilections. From a genetic point of view, RB mainly presents in two distinct clinical forms: the non-heritable form (majority), in which the tumors are unifocal and unilateral, usually presenting at an older age, and the heritable form (minority), usually presenting as multifocal, bilateral lesions at an early age. Although a well-known RB therapy paradigm is survival over vision preservation, increasingly multimodal and interdisciplinary therapies have been developed that target the tumor in its local environment before drastic options such as enucleation of the diseased eye must be considered. For many children, especially those with an unilaterally progressing disease, enucleation is the only reasonable rescue treatment option. Patients are usually assessed in accordance with the International Classification of Retinoblastoma (ICRB), allowing consensus regarding therapy, optimally preserving diseased eyes, and maximizing visual acuity (1). Through early diagnosis, the survival rates of affected children remain high in first-world countries. Late diagnosis is likely to be tied to metastatic spread and therefore associated with high morbidity and mortality, and it is a major problem in developing countries (2). Advancing therapies such as immune checkpoint inhibitors and oncogene-targeted drugs improve the management of various cancers such as melanoma, lung adenocarcinoma, squamous cell carcinoma, colon cancer, bladder cancer, and gastric cancer (3). Effective targeted therapy would be a promising new therapy approach and potentially could preserve visual acuity in some RB cases. To date, there are neither effective nor recognized targeted therapies for the treatment of RB reported in the current literature. Comparing anaplastic differentially graded RBs on a molecular level, undifferentiated retinoblastoma’s (URB’s) pathophysiology contributing factors to tumorigenesis can be explained. Research in the field of molecular biology remains challenging but is still very much needed for future advances in targeted therapies and molecular biomarkers, especially in RB research.

## 2 Material and methods

### 2.1 Sample population

Our study cohort consisted out of 21 enucleated retinoblastoma patients, divided into two subgroups. The subgroup was selected upon histopathological grading of anaplasia in these samples. In addition, there were two controls (non-tumorous tissue).

### 2.2 RNA extraction

One to three paraffin sections with a thickness of 7 μm per sample were deparaffinized with xylene prior to RNA extraction using the RNeasy FFPE kit (Qiagen, Hilden, Germany) in accordance with the manufacturer’s recommendations, albeit with slight adjustments. Total RNA concentrations were measured using a Nanodrop 1000 instrument (Thermo Fisher Scientific, Waltham, USA) (4).

### 2.3 nCounter CodeSet Design and Expression Analysis

Multiple genes involved in tumor- and inflammation-associated pathways were selected based on the current literature to screen for potential biological cues that could significantly differentiate RBs from the other lesions and also provide pathophysiological insights into these entities.

Gene expression patterns were screened for prognostic and predictive biomarkers using the NanoString’s nCounter digital gene expression analysis platform with its PanCancer Profiling panel, consisting of 770 genes and 30 reference genes. Hybridizations were performed using the high-sensitivity protocol on the nCounter Prep Station. Post-hybridization processing was performed using the nCounter MAX/FLEX System (NanoString), and the cartridge was scanned on the Digital Analyzer (NanoString). The cartridge was read with maximum sensitivity (555 FOV). A 100-ng sample was used as the input for each reaction.

### 2.4 NanoString data processing

NanoString data processing was carried out using the R i386 statistical programming environment (v4.0.3). Considering the counts obtained for the positive control probe sets, the raw NanoString counts for each gene were subjected to a technical factorial normalization, carried out by subtracting the mean count plus two times the standard deviation from the CodeSet inherent negative controls. Subsequently, biological normalization using the included RNA reference genes was performed. In addition, all counts with *p*>0.05 after a one-sided *t*-test versus negative controls plus two times the standard deviation were interpreted as not sufficiently expressed to overcome basal noise (5).

### 2.5 Statistical evaluation

Statistical analyses were carried out using the R i386 statistical programming environment (v4.0.2). Prior to exploratory data analysis, the Shapiro–Wilk test was applied to test for the normal distribution of each data set for ordinal and metric variables. The resulting dichotomous variables underwent either the Wilcoxon Mann–Whitney rank sum test (non-parametric) or the two-sided Student’s *t*-test (parametric). For the comparison of ordinal variables and factors with more than two groups, either the Kruskal–Wallis test (non-parametric) or an analysis of variance (ANOVA) (parametric) was used to detect group differences. Double dichotomous contingency tables were analyzed using Fisher’s exact test. To test the dependency of ranked parameters with more than two groups, Pearson’s chi-squared test was used. Correlations between metrics were tested by applying Spearman’s rank correlation test and Pearson’s product–moment correlation testing for linearity. A basic quality control of the processed data was performed by mean versus variance plotting to find outliers in the target or sample levels. True differences were calculated by a correlation matrix analysis. To further specify the different candidate patterns, both unsupervised and supervised clustering, in addition to principal component analysis, were performed to overcome commonalities and differences. The sensitivity and specificity of markers were determined from receiver operating characteristic (ROC) curves, illustrating their ability to discriminate between the studied groups. The bootstrap procedure (1,000 iterations) was used for internal validation of the estimates in the ROC analyses. The best candidate genes were selected and binarized (0, 1; with 1 equaling a better chance of an event) by their respective cut-offs and finally summarized. The resulting scores were compared with respect to sensitivity and specificity. The probability for each entity was determined using the non-linear (weighted) least-squares estimates of the parameters of a non-linear fitted regression model (6, 7). Adaptations of profiles for diagnostic purposes were modeled with a supervised machine learning tool, conditional inference trees (CTrees), as implemented in the “party” library of R (8) using leave-one-out cross-validation. CTrees are a non-parametric class of regression trees leading to a non-parametric class of tree-structured regression models embedding a conditional inference procedure, applicable to all kinds of regression problems, such as nominal, ordinal, numeric, and censored, in addition to multivariate response variables and arbitrary measurement scales of the covariate (8). Owing to the multiple statistical tests, the *p*-values were adjusted by using the false discovery rate (FDR). The level of statistical significance was defined as *p* ≤ 0.05 after adjustment.

## 3 Results

### 3.1 Study population

**Figure 1:**
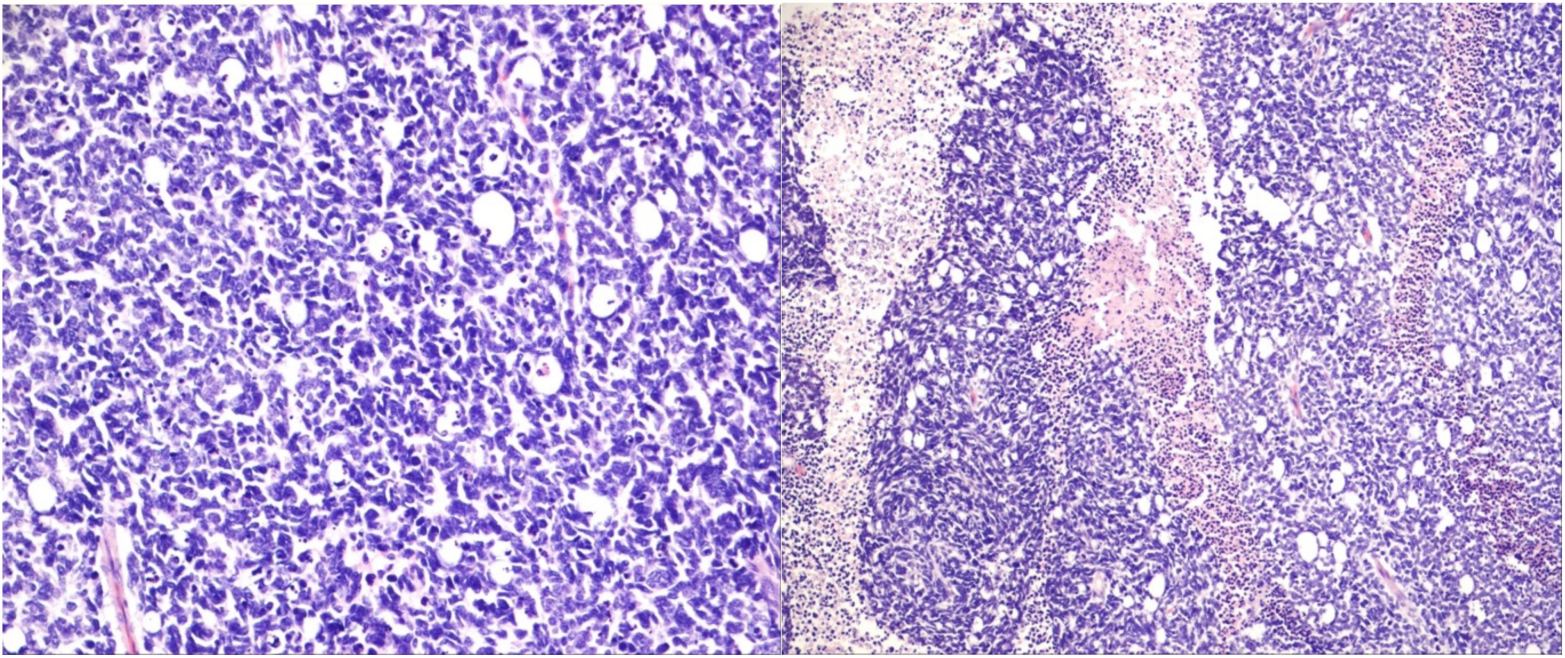
Undifferentiated retinoblastoma with proposed high-risk features. Left: histologic evaluation shows small tumor cells with scant cytoplasm and condensed chromatin; right: prominent necrosis. (Hematoxylin and eosin staining: left, 20×; right, 10×).

**Figure 2:**
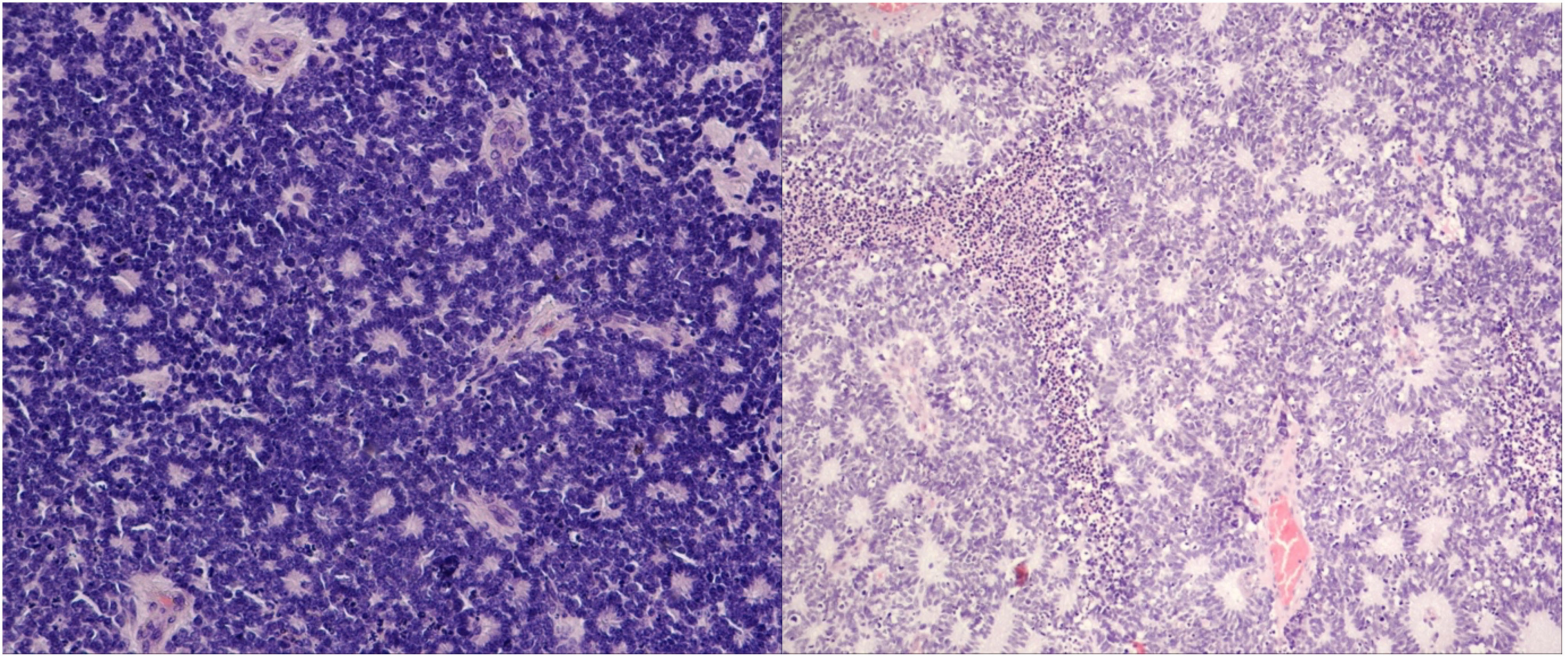
Differentiated retinoblastoma. Left: photoreceptor differentiation (Flexner–Wintersteiner and Homer Wright rosettes); right: enlargement of nuclei similar in size to moderate anaplasia, pleomorphism (angular, rhomboid, or fusiform), cell wrapping, numerous mitotic figures, and necrosis (hematoxylin and eosin staining: left, 40×; right, 10×).

The mean age of the 21 patients was 1.7 years, and 10 patients (47.6%) were female. The median age at diagnosis was 14 months (range: 2–50 months). Additional clinical characteristics are included in **Table 1** and **Supplementary Data 1**. We analyzed biopsy specimens from 21 RB patients, and these were divided into two subgroups: 11 URBs (**Figure 1**) and 10 differentiated retinoblastomas (DRBs) (**Figure 2**)

**Table 1:** Clinical characteristics of patients stratified by molecular subtype.

Characteristics	URBs	DRBs	*p*-value	Statistical test
Patients, *n* (%)	11 (52)	10 (48)		
Sex, *n* (%)				
Women	2(25)	6(75)	0.04	Chi-square
Men	9 (70)	4(30)		
Laterality, *n* (%)				
Unilateral	5(45)	6(55)	0.5051	Chi-square
Bilateral	6 (60)	4 (40)		
Age at diagnosis (months)				
Median (range)	14 (4–50)	10.5 (2–45)	0.436	Mann–Whitney test
<18, *n*	8	6	0.2	Kruskal–Wallis test
18–36, *n*	1	2		
>36, *n*	2	2		
Tumor stage:N*, *n*				
N0	3	4	0.06	Kruskal–Wallis test
N1	6	6		
N2	2	0		
C*, *n*				
C0	7	7	0.2	Kruskal–Wallis test
C1	2	2		
C2	2	1		
RB1 germline mutation, *n* (%)				
Yes	6 (75%)	2(25%)	0.1827	Fisher’s exact test
No	5 (38.46%)	8 (61.54%)		

*The TNM classification for retinoblastoma, which is developed by the American Joint Committee on Cancer (AJCC).

### 3.2 Anaplastic grade

The degree of differentiation in RB tumors was mostly defined by the development of fleurettes and rosettes. Samples were graded accordingly by a pathologist.

### 3.3 Expression analysis

**Figure 3:**
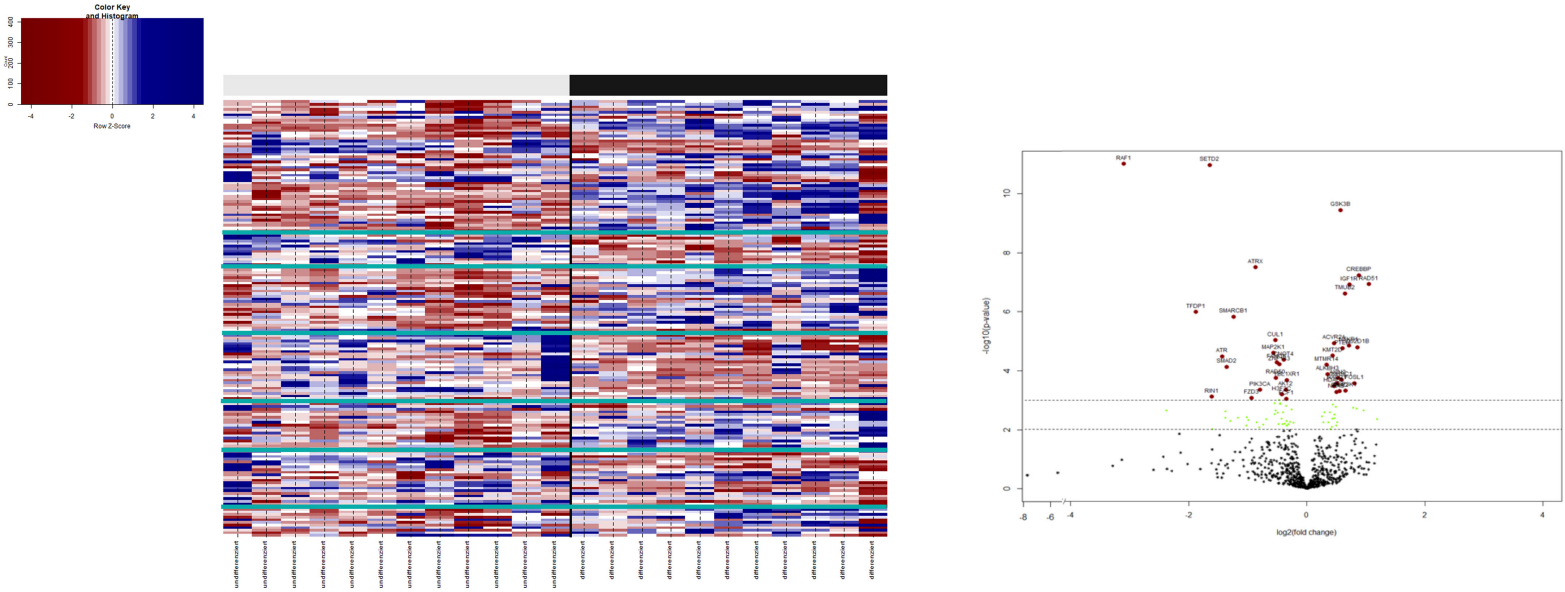
3.1: Heatmap: on the bottom x-axis, each investigated patient with its entity identifier is depicted. The upper x-axis shows the binary clusters of the entity groups with respect to their histopathological stratification. The virtual y-axis depicts 770 differentially expressed RNA targets. The color key on the left indicates the expression ratio, in which highly expressed genes are indicated in blue and poorly expressed genes in red. 3.2: Volcano plot illustrating the differential gene expression between URB and DRB. A total of 166 out of 770 differentially expressed genes with an adjusted *p*-value of < (0.05) are shown. In total, 38 genes (41.8%) show expression only or in a much stronger manner in DRB (left side), whereas 53 (58.2%) genes present with overexpression in URBs (right side). Red dots indicate a highly significant association and green dots indicate a significant association identified by explorative data analysis using either the Wilcoxon Mann–Whitney rank sum test (non-parametric) or the two-sided Student’s *t*-test (parametric).

Gene expression analysis was successful in all samples: 21RB samples and two control samples. After biological and technical normalization, 726 (94.3%) out of 770 genes were identified as transcripts with relevant gene expressions. We compared the transcriptomes of the two subtypes: 166 (22.9%) genes were found to be differentially expressed between subtypes (**Figure 3**), with an adjusted *p*-value of <0.05.

### 3.4 Gene set enrichment analysis

To identify biological mechanisms (pathways and biological functions) affected by the different expression patterns of immune genes in URB and DRB, a gene set enrichment analysis (GSEA) was performed.

### 3.5 Mitogen-activated protein kinase signaling pathway

The following main conductors for the classical mitogen-activated protein-kinase (MAP-K) pathway were elevated in the URB samples: nerve growth factor (NGF), neutrophin 3/4 (NT3/4) (9), epidermal growth factor (EGF), and platelet-derived growth factor (PDGF) and its downstream ligand platelet-derived growth factor receptor (PDGFR). Throughout the pathway, RAS, MEKK1 (10), FASL (11), and P53 were also elevated in these samples, all of which contribute to apoptosis.

### 3.6 The PI3K/AKT/mTOR signaling pathway

The main conductors to the activation of the Pi3K-Akt signaling pathway, GF, and extracellular matrix (ECM), were elevated in the DRB samples. Also, ECM’s downstream ligands ITG-A and B, together with the common pathway PI3K, were elevated in these samples. Most ligands responsible for cell survival and DNA repair—Bcl-xL, Bcl-2, and MEK—were also elevated in the DRB samples, whereas ligands mostly responsible for cell cycle progression coming from the PI3K-Akt signaling pathway—CDK, Cyclin, and Myc—were elevated in the URB samples.

### 3.7 Cell-cycle progression and inhibition

**Figure 4:**
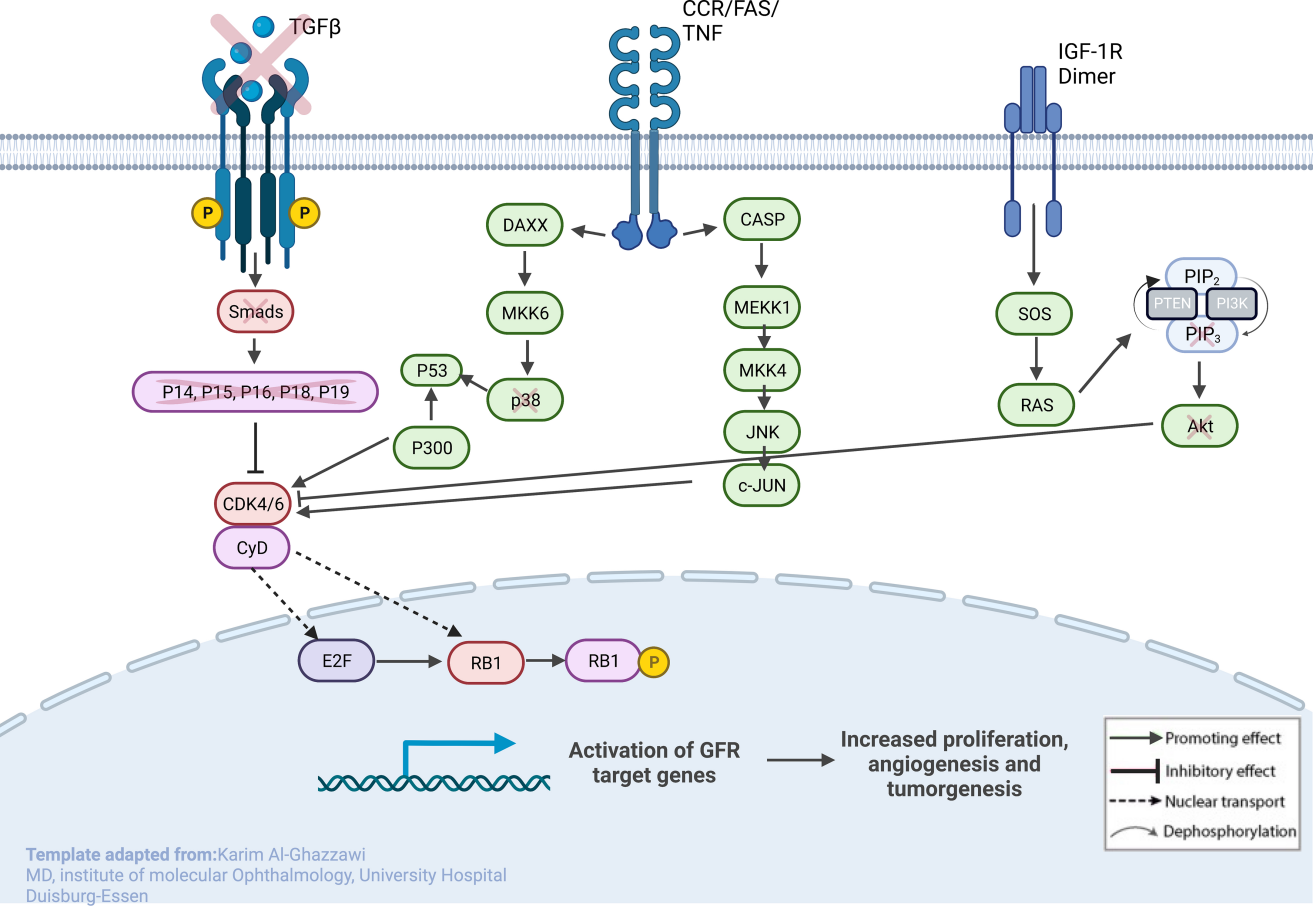
Sketch of the investigated genes that correlate with explicit molecules that contribute to tumorigenesis in RB, leading to predominant undifferentiated phenotypes. From left to right: (1) loss of TGF-β and SMADs in URB samples, leading to the missing inhibitory effects of p16, p15, p18, and p19 on CycD and CDK4,6; (2) CCR/FAS/tumor necrosis factor (TNF) receptor with main conductors CASP and DAXX being overexpressed in URBs; and (3) IGF-1R with downstream ligands SOS and RAS being overexpressed in URB samples. Together, this will ultimately lead to proliferative signals in the nucleus with invasiveness, prominent angiogenesis, and tumorigenesis.

URBs showed an upregulation of P300, P53, CyD, CDK4,6, and E2F1,2,3 when compared with DRBs, thereby contributing to cell-cycle progression in this configuration (**Figure 4**). Healthy cells experiencing oncogenic “stress” use the P53 pathway to exit the cell cycle and induce apoptosis; physiologically, it induces cell-cycle arrest to create time for DNA repair to restore genome stability. One hallmark of cancer is the evasion of apoptosis; achieving this continues proliferation, which can be achieved through P53 mutation and inactivation. DRBs showed upregulation in transforming growth factor-β (TGF-β) and SMADs2,3, together with their downstream ligands P15 and P19.

## 4 Discussion

Current understandings of disease initiation in both hereditary and sporadic forms of RB tumors are linked to a loss of function in the RB gene (RB1) (12). Mutations leading to the aberrant function of the RB tumor suppressor protein (pRB) have been found not only in retinoblastoma cancer and cell lines but also in various other tumors, such as osteosarcoma, adenocarcinoma, small cell lung cancer, breast cancer, and prostate cancer (13). In this study, we examined differentiated and undifferentiated RB samples. The degree of differentiation in RB is determined by the development of rosettes and fleurettes. Mendoza et al. examined clinical and pathologic findings in patients who underwent primary enucleation for RB. The study assessed the grade of anaplasia and differentiation regarding the presence or absence of high-risk features (14). What is debatable is whether or not prognosis in RB is concluded from differentiation, as studies have shown conflicting results (15, 16). Poorly differentiated tumors have been associated with high-risk features, especially massive choroidal invasion (17). As genetic profiling studies of RBs keep increasing (18–20), four studies based on gene expression profiling delivered partially conflicting results regarding RB subtypes (19, 21, 22). The aims of this study included differentiating RB on a molecular level by focusing on possible docking points for targeted therapies.

### 4.1 Pi3Akt and MAPK signaling pathways in retinoblastoma

**Figure 5:**
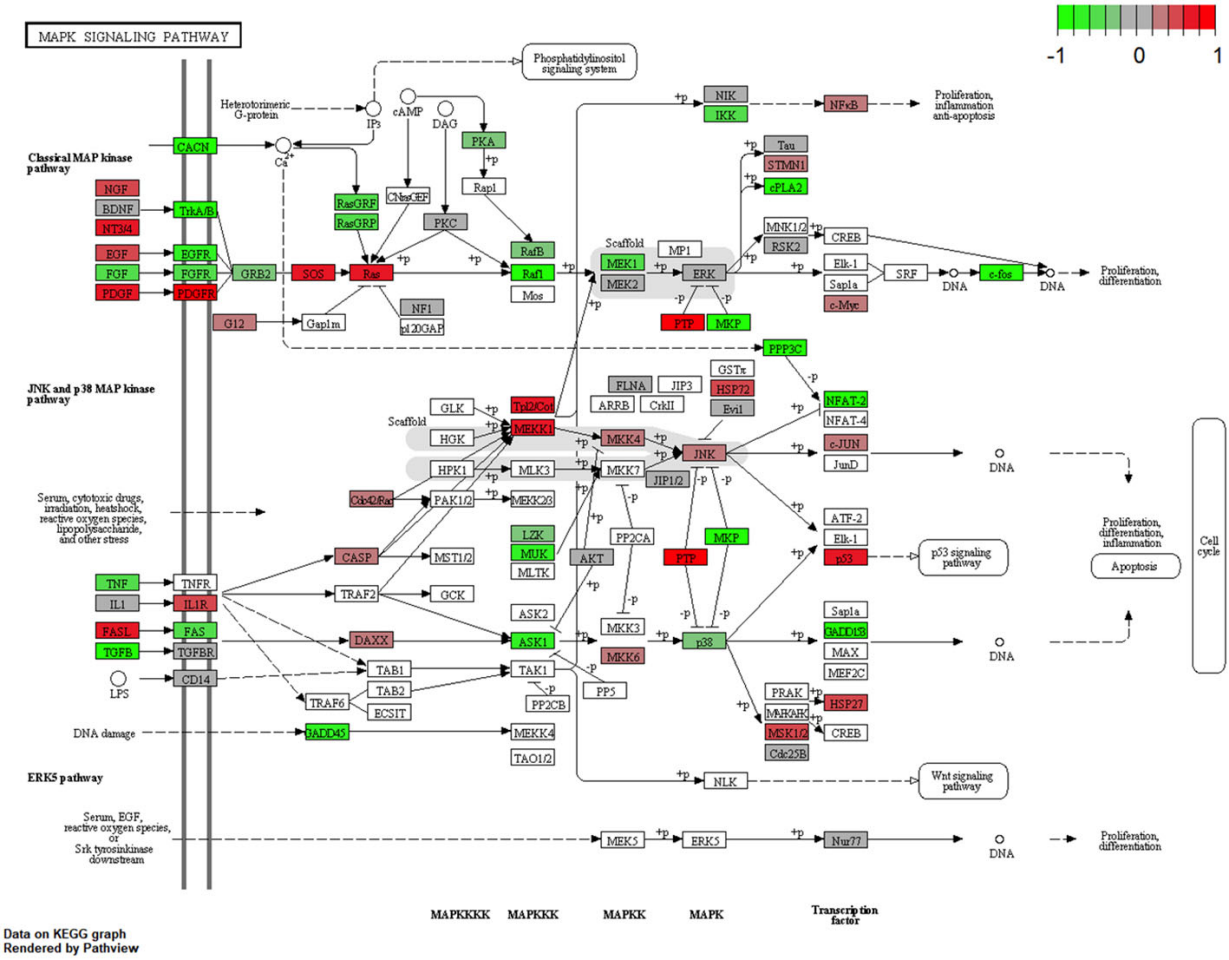
Expression of different pathways involved in the MAP-K signaling pathway. Pathways marked in red were overexpressed (1), and green pathways were downregulated (−1) when comparing URBs with DRBs. The main conductors for the classical MAP-K pathway, NGF, NT3/4, EGF, and PDGF, and its downstream ligand, PDGFR, were elevated in the URB samples.

**Figure 6:**
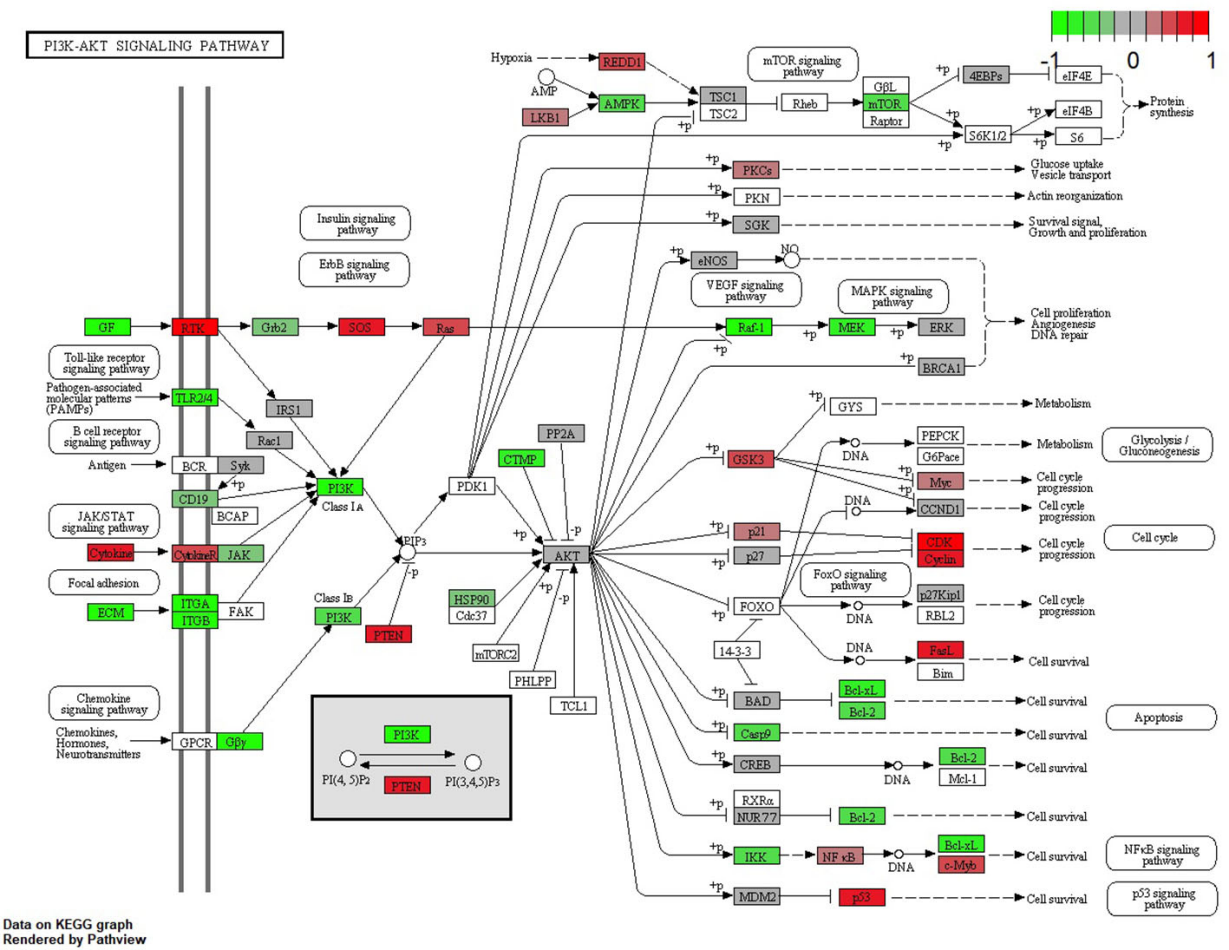
Expression of different pathways involved in the PI3K-AKT signaling pathway. Pathways marked in red were overexpressed (1) and green pathways were downregulated (−1) when comparing URBs with DRBs. The main conductors to the activation of the Pi3K-Akt signaling pathway, GF and ECM, were elevated in the DRB samples. In addition, ECM downstream ligands ITG-A and B, together with the common pathway PI3K, were elevated in these samples.

**Figure 7:**
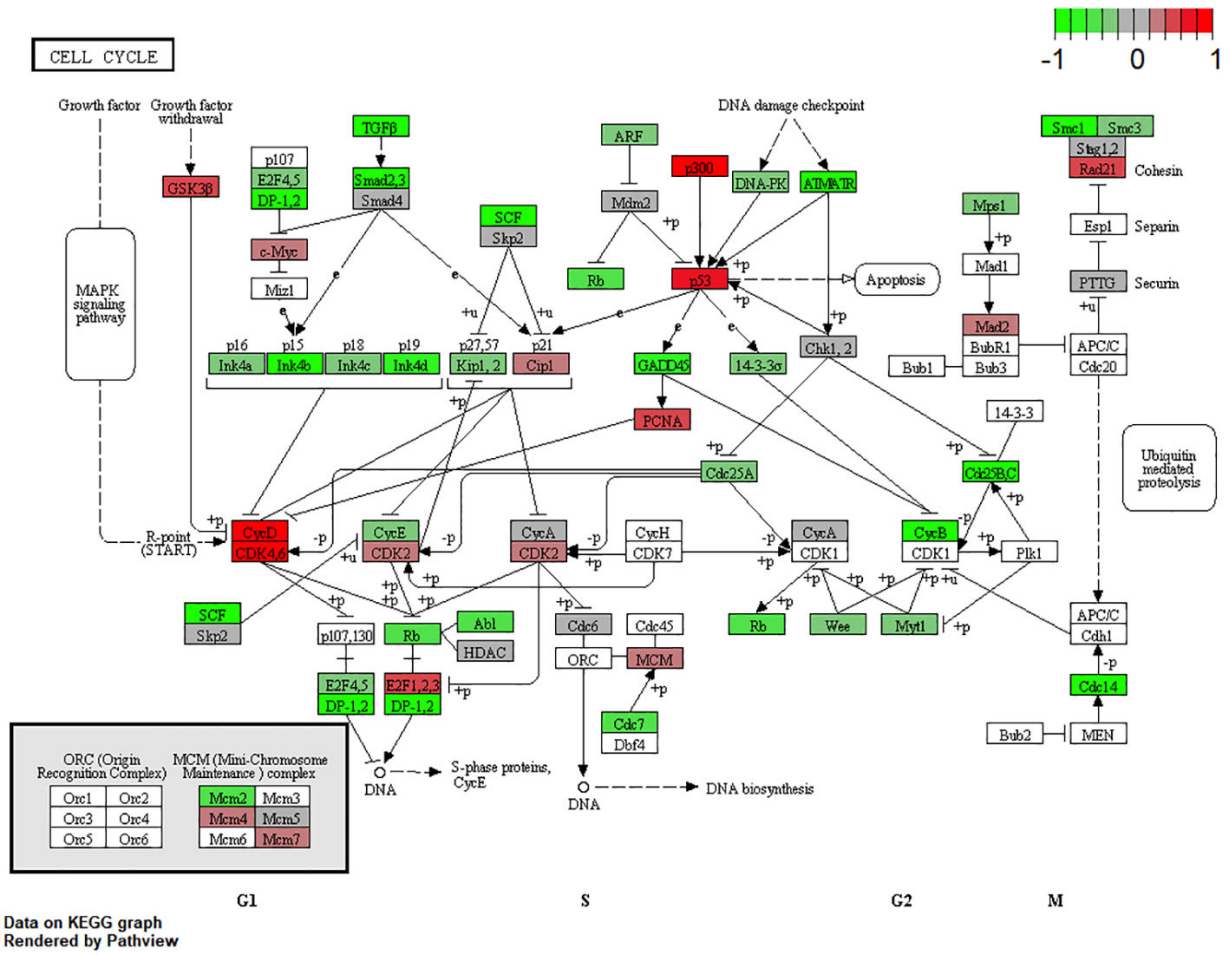
Expression of different pathways involved in the cell-cycle signaling pathway. Pathways marked in red were overexpressed (1), and green pathways were downregulated (−1) when comparing URBs with DRBs. URBs showed an upregulation of P300, P53, CyD, CDK4,6, and E2F1,2,3 when compared with DRBs. DRBs showed an upregulation of TGF-β and SMADs2,3, together with their downstream ligands P15 and P19.

This study examined different agents that regulate cell-cycle progression, inhibition, and apoptosis through pathways such as MAPK signaling (**Figure 5**), Pi3Akt signaling (**Figure 6**), and general cell-cycle control (**Figure 7**). On a molecular level, cell-cycle inhibition and progression are regulated by different pathways, such as MAPK signaling and Pi3Akt signaling. Both pathways are very complex, with interconnected signaling cascades, and are still not fully understood. Physiologically, both pathways are initiated by extracellular and intracellular stimuli in the form of proteins functioning as cytokines and binding other factors to form kinases, leading to regulated activation and inhibition. In a simplified scheme, the result of activating the MAPK pathway is increased cellular proliferation, whereas activating the PI3K pathway stimulates protein synthesis and inhibits apoptosis (23, 24). In a previously published study by Liu et al., a similar analysis of RB samples was performed; however, they chose to approach the problem distinctly (19). A high-risk retinal subtype was defined, converging with recurring genetic alliterations and less histopathologically identifiable differentiation. We therefore see a validation of our study results. We observed an increased signal for activators of the classical non-canonical MAP-Kinase pathway, NGF, NT3/4, EG, F, and PDGF, in the URB samples.

The Pi3K Akt signaling pathway (phosphatase and tensin homolog) subsequently dephosphorylates PIP3 to PIP2, an event that is a key element in regulating the pathway (24). Also, the URB samples showed activation for this enzyme. PIP2 is therefore more likely to be present in the cytoplasm, leading to the decreased catalytic activity of AKT, together with the effector ligands Bcl-xL and Bcl-2, causing cell progression instead of apoptosis (25, 26). Although GSK-3b and even p21 showed higher base expression levels in the URB group than in the DRB group, we considered this to be a feedback loop-like response, and this did not indicate the inhibition of the pathway. This undermined the stronger expression of CDK4/6 in addition to CDK2 overexpression and E2F activation; moreover, the loss of TGF-b and the SMAD-based induction of apoptosis and cell cycle stasis did not support this finding. One of the most altered pathways in cancer morphologies (27), the “RTK-RAS pathway”, which cross-talks within the MAPK and the Pi3k-Akt signaling pathways (28, 29), showed significant differences when comparing both cohorts. URBs showed overexpression for SOS and RAS; in comparison, DRBs had elevated PI3K signals.

### 4.2 TGF-β

TGF-β signaling is one of the impaired pathways in RB. In the early stages of cancer, TGF-β inhibits cell-cycle progression and promotes apoptosis by exhibiting tumor-suppressive effects. However, in the late stages, TGF-β exerts tumor-promoting effects, increasing tumor invasiveness and metastasis. Physiologically, TGF-β acts as a tumor suppressor through the inactivation of TGF-β receptors and SMADs. The downregulation of receptors and increased expression of TGF-β signaling inhibitors have been reported in human cancers (30). URB samples showed significantly decreased levels of both TGF-β and its downstream ligands, SMAD 2, and 3, when compared with DRBs. Ultimately, the missing inhibitory effects of p16, p15, p18, and p19 on CycD and CDK4,6 in URBs will lead to proliferative signals in the nucleus (**Figure 4**). Several therapeutic tools such as TGF-β antibodies, have been tested for their anti-tumor effects. Antisense oligonucleotides and small inhibitor molecules of TGF-β receptor-1 (TGF-βR1) have shown potential inhibitory effects on TGF-β signaling (31).

### 4.3 E2F

In healthy cells, RB suppresses transcription by binding to the chromatin-remodeling proteins BRG and histone deacetylases (HDACs). Furthermore, it regulates the transition from the G1 to the S phase of the cell cycle by binding to the E2F family of transcription factors. In RB cells, dysfunction of the RB protein leads to the constant activity of the E2F transcription domains. The cell cycle is therefore more likely to halt during the late G1/early S phase transition, leading to a continuous instigation of the cell cycle and tumor growth (32, 33). A recent study proposed that E2F is part of the E2F1/CKS2/PTEN signaling axis as a key regulator of malignant phenotypes in RB (34). We hypothesized that this tumor growth is accelerated and mediated by a constant loop-like transcription or translation regulation of effector genes of the growth factor family: NGF, NT3/4, EGF, and PDGF.

### 4.4 RB protein

RB* is a tumor-suppressor gene in Retinoblastoma and in many other cancers as well. Periodic phosphorylation of the RB protein makes cell division cycles possible. Studies have detected activated (hypophosphorylated) forms of the protein in G1, which inactivates (hyperphosphorylates) in late G1 and remains in this state throughout the S phase through mitosis. The RB protein acts as a tumor suppressor in the hypophosphorylated (active) state by restricting proliferation (35, 36). Cyclin-dependent kinases 4 and 6 (CDK4 and CDK6) play key roles in cell proliferation, in which they help to drive the progression of cells into the S phase of the cell cycle (37). In various cell models, it has already been shown that CDK4 and CDK6 are fundamental drivers of the cell cycle and are required for the initiation and progression of various malignancies (38).

The PTEN mutational status is known to be a crucial regulator and progressor in RB carcinogenesis. It induces various feedback loops, resulting in strong overexpression of the impaired catalytic protein activity. Moreover, clinically, the PTEN mutational status affects the response to combined therapy based on MEK and mTOR inhibitors in cancer (39); this should be taken into consideration when looking at individualized RB therapies in the future.

Circulating chemokines such as tumor necrosis factor (TNF) and interleukin (IL)-1 can influence the surrounding tumor tissue. For example, TNFs probably represent a danger signal in response to neoplastic tissue damage to rid the organism of premalignant tissue or to promote wound healing (40). A possible relationship between TNF-α overexpression and RB malignancy has been previously discussed by Pellestor et al. (41). The JNK signaling pathway, derived from MEKK/MKK signaling, is a possible target for these stimuli (42), forming appropriate cellular responses in the form of proliferation, differentiation, and apoptosis. The pathway has been described as being active for both apoptosis and differentiation, depending on the conditions (43). It has been described as being activated by environmental oxidative stress and inflammatory stimuli (44). JNK and its downstream cascade, c-JUN, were consistent in the URB samples. Transcriptional effectors of this cascade can modify nuclear DNA with proliferative signals in tumor cells (45). The molecular characterization of RB patient samples is currently being performed on tumor samples obtained from enucleation. The analysis of RB using liquid biopsy (46) could provide a more comprehensive picture of the disease. Cell-free DNA aliquots from blood samples could potentially be used to optimize RB treatment and prognosis.

The limitations of this study include the absence of non-tumoral retinal control samples. The harvesting of a non-tumoral healthy retina as a control is challenging, as there is no medical incentive to retrieve healthy retinal tissue from comparable patients. The use of detached retinal tissues from vitrectomies combined with retinectomies could be an option as a control; however, even this retinal tissue may show alterations due to the underlying disease and may not resemble a healthy retina. Another limitation is the unknown mutational status. To overcome this limitation, a thorough literature review was commenced. The gene panel used, with over 800 targets, is limited in size; however, the screened genes are part of the most important expression targets leading to carcinogenesis. A whole-transcriptome evaluation could investigate many more targets; however, it could also draw attention to faulty side players. With our current approach, we are therefore dealing with less background noise and taking only prominent trails into consideration. Based on regulatory feedback loops leading to increased expressions of affected tumor-suppression genes in our study, together with a detailed literature review, we have found supporting arguments for our hypothesis. In this study, we observed possible molecular targets through an expression analysis. Discriminative markers should also be examined in a traditional format to identify underlying relevant proteins, as changes may be transient, post-translational, and/or in non-target organs. Contributing genes to pathogenesis could be examined in a knock-down study to distinguish the cause and effect of specific genes. Despite obvious limitations such as low case numbers and limited transcriptomic cutouts, we are highly convinced of our general results and hypothesis, as the overall observed results support our hypothesis regardless of the complexity of further pathways and molecular mechanisms. Through the observed clinical behavior regarding outcome (metastasis and survival) and the biological risk features that resulted in the phenotypical expression of malignant transformation, we clearly see evidence for our proposed hypothesis.

## 5 Conclusion

From a biological point of view, there is a remarkable difference between both entities examined: the examined tissues showed various differences regarding key cancer regulatory pathways. The presence of growth factors through activators of the classical non-canonical MAP-Kinase pathway, NGF, NT3/4, EGF, and PDGF, was significant in URB samples when compared with DRBs. In contrast, DRBs were consistent with activators of the Pi3k-AKT signaling pathway altogether with DNA-repair mechanisms contributing to the cell cycle progression instead of the apoptotic conductors in URB samples. We are not sure where the loop-like induction of these pathways begins, but we are certain that it has regulatory effects on cancer formation and progression. The identification of molecular markers pointing to high-risk features should redefine further studies of this cancer, to include the identification of more specific treatments and improvements in diagnosis and prognosis.

## Data availability statement

The original contributions presented in the study are included in the article/**Supplementary Materials**. Further inquiries can be directed to the corresponding author/s.

## Ethics statement

The studies involving humans were approved by the Ethics Commission of the University of Essen. The studies were conducted in accordance with the local legislation and institutional requirements. The participants provided their written informed consent to participate in this study.

## Author contributions

Conceptualization, FM, ST; methodology, MW, KA-G, and FM; software, FM, MW; validation, KA-G, and FM; formal analysis, EB, KA-G, and FM; investigation, KA-G, PK, JL, EB, and FM; resources, KA-G, SD, TK, JL, ST, EB, MW, NB and FM; data curation, FM, and KA-G; writing—original draft preparation, FM, KA; writing—review and editing, EB, KA-G, NB, FM, TK, JL,SD, ST, PK, and MW; visualization, FM, ST, KA-G; supervision, FM, PK, ST, project administration, MW, TK, SD, KA-G FM;NB funding acquisition, FM. All authors have read and agreed to the published version of the manuscript.
