# Erratum: PDGF, NGF, and EGF as main contributors to tumorigenesis in high-risk retinoblastoma

**DOI:** 10.3389/fonc.2023.1339187

**Published:** 2023-12-07

**Authors:** Frontiers Production Office

**Affiliations:** Frontiers Media SA, Lausanne, Switzerland

**Keywords:** retinoblastoma - epigenetics, retinoblastoma, PDGF = platelet-derived growth factor, MAPK/ERK2 pathway 1, pediatric cancer and oncology, biomarkers, targeted therapy, growth factors (angiogenesis factors)

Due to a production error, section **3.2 Anaplastic grade** included two empty subsections: **3.2.1 Undifferentiated retinoblastoma** and **3.2.2 Differentiated retinoblastoma.** These subsections have been removed.

The publisher apologizes for this mistake. The original version of this article has been updated.

